# Radiation Effects on the Flow of Powell-Eyring Fluid Past an Unsteady Inclined Stretching Sheet with Non-Uniform Heat Source/Sink

**DOI:** 10.1371/journal.pone.0103214

**Published:** 2014-07-29

**Authors:** Tasawar Hayat, Sadia Asad, Meraj Mustafa, Ahmed Alsaedi

**Affiliations:** 1 Department of Mathematics, Quaid-i-Azam University, Islamabad, Pakistan; 2 School of Natural Sciences (SNS), National University of Sciences and Technology (NUST), Islamabad, Pakistan; 3 Department of Mathematics, Faculty of Science, King Abdulaziz University, Jeddah, Saudi Arabia; Northwestern Polytechnical University, China

## Abstract

This study investigates the unsteady flow of Powell-Eyring fluid past an inclined stretching sheet. Unsteadiness in the flow is due to the time-dependence of the stretching velocity and wall temperature. Mathematical analysis is performed in the presence of thermal radiation and non-uniform heat source/sink. The relevant boundary layer equations are reduced into self-similar forms by suitable transformations. The analytic solutions are constructed in a series form by homotopy analysis method (HAM). The convergence interval of the auxiliary parameter is obtained. Graphical results displaying the influence of interesting parameters are given. Numerical values of skin friction coefficient and local Nusselt number are computed and analyzed.

## Introduction

The study of boundary layer flow and heat transfer over a stretching sheet has gained considerable attention due to its numerous practical applications such as paper production, hot rolling, drawing of plastic films, annealing and tinning of copper wires and metal spinning. Wang [1] proposed the problem of unsteady two-dimensional boundary layer flow of liquid film on unsteady stretching sheet. Later Andersson et al. [2] extended Wang's problem for heat transfer effects by considering time-dependent wall temperature. Further Elbashbeshy and Bazid [3] investigated the thermal boundary layer in the time dependent flow (occupying a semi-infinite domain) over an unsteady stretching surface. Ishak et al. [4] studied heat transfer over an unsteady stretching permeable surface with prescribed wall temperature. Radiation effects on the flow and heat transfer over an unsteady stretching surface with internal heat generation were analyzed by Abd El-Aziz [5]. Shateyi and Motsa [6] examined the radiation effects on the time dependent flow of liquid film on unsteady stretching sheet with heat and mass transfer. They obtained an analytic solution of the resulting problem by Chebyshev pseudo-spectral collocation method. Tsai et al. [7] investigated the flow and heat transfer over an unsteady stretching surface with non-uniform heat source. Mukhopadhyay [8] numerically analyzed the flow over unsteady permeable stretching sheet with variable suction and time-dependent surface temperature. In this study, the fluid with variable viscosity and variable thermal conductivity was taken into consideration. Analytic solutions for radiation effects on mixed convection flow of Jeffrey fluid and heat transfer past an unsteady stretching sheet were provided by Hayat et al. [9]. Three dimension elastico-viscous flow over an unsteady stretching sheet has been discussed by Hayat et al. [10]. Mukhopadhyay [11] extended the work [8] for flow near a stagnation-point with variable free stream. MHD stagnation-point flow of an electrically conducting Casson fluid past an unsteady stretching surface was explored by Bhattacharyya [12]. Yang and Baleanu [13] investigated the fractal heat conduction problem. They solved by using local fractional variation iteration method. Yang et al. [14] presented local fractional Fourier series solutions for non-homogeneous heat equations arising in fractal heat flow with local fractional derivative.

It has now been widely recognized that in industrial and engineering applications, non-Newtonian fluids are more suitable than Newtonian fluids. Due to the flow diversity in nature, the rheological features of non-Newtonian fluids cannot be captured by a single constitutive relationship between stress and shear rate. For this reason, a variety of non-Newtonian fluid models (exhibiting different rheological effects) are available in the literature [15], [16]. Amongst those is the Powell-Eyring fluid [17] which although mathematically complex has tendency to describe the flow behavior at low and high shear rates. It can be used to formulate the flows of modern industrial materials such as powdered graphite and ethylene glycol. Unidirectional flow of Powell-Eyring fluid between parallel plates with couple stresses was studied by Eldabe et al. [18]. Pulsatile flow of Powell-Eyring fluid was examined by Zueco and Beg [19]. Homotopy perturbation analysis of slider bearing lubricated with Powell-Eyring fluid was presented by Islam et al. [20]. Three-dimensional flow of Powell-Eyring fluid past a wedge was discussed by Patel and Timol [21]. Boundary layer flow of Powell-Eyring fluid over a moving flat plate was analyzed by Hayat et al. [22]. Recently steady flow of Powell-Eyring fluid over an exponentially stretching sheet was numerically investigated by Mushtaq et al. [23]. It has been noted that literature is scarce for unsteady flow of Powell-Eyring fluid. To our information, the flow and heat transfer of the Powell-Eyring fluid thin film over an unsteady stretching sheet are examined by Khader and Megahed [24]. Impact of uniform suction/injection in unsteady Couette flow of Powell-Eyring fluid is explored by Zaman et al. [25].

The present work considers the boundary layer flow of Powell-Eyring fluid over an unsteady stretching sheet. The stretching sheet is considered inclined. In addition the effects of radiation and non-uniform heat source/sink are also taken into account. Radiative heat transfer in the boundary layer flow is very important from application point of view, because the quality of the final product is very much dependent on the rate of heat transfer of the ambient fluid particles. Such radiative effects are also important in many non-isothermal cases whereas the heat generation/absorption in moving fluids is significant in the applications involving heat removal from nuclear fuel debris, underground disposal of radioactive waste material, storage of food stuffs, dislocating of fluids in packed bed reactors and several others. Similar situations prevail during the manufacture of plastic and rubber sheets where it is often necessary to blow a gaseous medium through the not-yet solidified material, and where the stretching force may be varying with time. The dimensionless mathematical problems are solved analytically by homotopy analysis method (HAM) [26]–[40]. Homotopy analysis method (HAM) is one of the most efficient methods in solving different type of nonlinear equations such as coupled, decoupled, homogeneous and non-homogeneous. Many previous analytic methods have some restrictions in dealing with non-linear equations. For illustration, in contrast to perturbation method, HAM is independent of any small or large parameters and or the existence of auxiliary parameter provides us with a simple way to control and adjust the convergence region which is a main lack of previous techniques. Also, HAM provides us with great freedom to choose different initial guesses to express solutions of the nonlinear problem. Numerical values of wall velocity and temperature gradient are computed and examined.

## Mathematical Formulation

**Figure 1:**
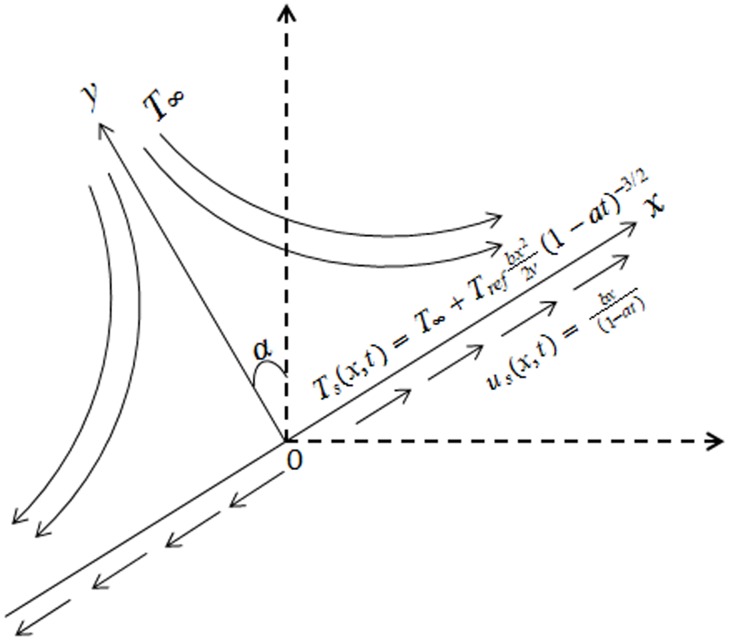
Physical model and coordinate system.

We consider unsteady two-dimensional incompressible flow of Powell-Eyring fluid past a stretching sheet. The sheet makes an angle 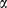 with the vertical direction. The x - and y-axes are taken along and perpendicular to the sheet respectively. In addition the effects of thermal radiation and non-uniform heat source/sink are considered (see Fig. 1). The Cauchy stress tensor in Powell-Eyring fluid is given by [17]:
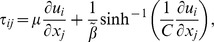



where μ is the viscosity coefficient, β and C are the material fluid parameters. The boundary layer equations comprising the balance laws of mass, linear momentum and energy can be written as [19]–[26]

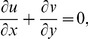
(1)

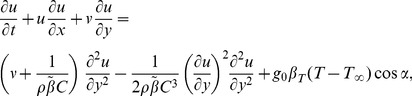
(2)

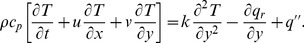
(3)


In the above expressions t is the time, 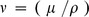 is the kinematic viscosity, k is the thermal conductivity of the fluid, 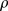 is the fluid density, T is the fluid temperature, 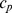 is the specific heat, 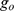 is the acceleration due to gravity, 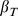 is the volumetric coefficient of thermal exponential, 
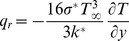
 [36]–[38] is the linearized radiative heat flux, 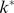 is the mean absorption coefficient, 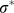 is the Stefan-Boltzmann constant, 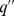 is the non-uniform heat generated 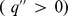 or absorbed 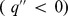 per unit volume. The non-uniform heat source/sink, 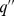 is modeled by the following expression [39]–[40].
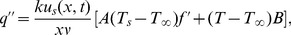
(4)


in which A and B are the coefficient of space and temperature-dependent heat source/sink, respectively. Here two cases arise. For internal heat generation A>0 and B>0 and for internal heat absorption, we have A<0 and B<0.

The surface velocity is denoted by 
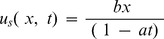
 whereas the surface temperature 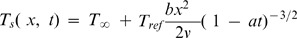. Here b (stretching rate) and a are positive constants having dimension time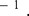 Also 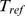 is a constant reference temperature. We note that the temperature of stretching sheet is larger than the free stream temperature 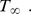


The boundary conditions are taken as follows:
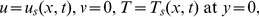
(5)

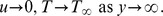



Introducing
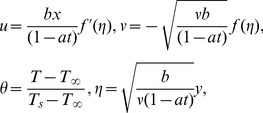
(6)


Eq. (1) is identically satisfied and Eqs. (2)–(5) become
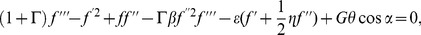
(7)

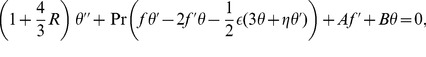
(8)

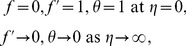
(9)


where prime denotes differentiation with respect to 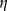, 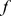 is the dimensionless stream function, 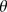 is the dimensionless temperature and the dimensionless numbers are 
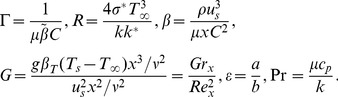
(10)


Here Г and 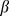 are dimensionless material fluid parameters, R is the radiation parameter, 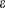 is the unsteady parameter and 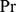 is the Prandtl number.

Local Nusselt number 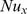 is defined as
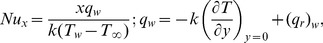
(11)

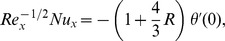



where 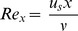 is the local Reynolds number.

## Solution Methodology

Most of the problems occurring in the field of science and engineering are non-linear. Specifically most of the problems encountered in fluid mechanics are highly non-linear. To find the exact solution of these non-linear problems is very difficult and some times even impossible. Thus several numerical and analytical techniques have been developed to solve such kind of problems. Among these HAM is the most used analytical technique. Convergent series solutions of non-linear equations are obtained.

### Homotopy analysis method

HAM was proposed by means of homotopy, a fundamental concept of topology. Two functions are said to be homotopic if one function can be deformed continuously into the other function. If 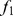 and 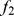 are two continuous maps from the topological space X into the topological space Y then 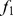 is homotopic to 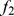 if there exist a continuous map F
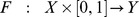



such that for each x
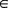
X
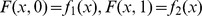



The map F is called homotopy between 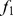 and 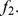


It should be noted that there is a great freedom to choose initial guess and auxiliary linear operator £. Beside such a great freedom there are some fundamental rules which direct us to choose the mentions parameters in more efficient way. Therefore, initial guesses for the velocity and temperature fields are taken in such a way that they satisfy the boundary conditions given in Eq. (9). And we choose linear operator specified in Eq. (13) that must satisfy the properties given in Eq. (14).
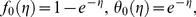
(12)

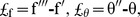



subject to the properties 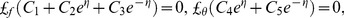
(13)


where 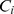 (i = 1–5) are the constants.

The deformation problems subjected to zeroth order
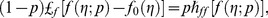
(14)

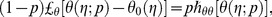
(15)

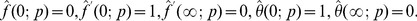
(16)


If p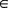 [0,1] indicates the embedding parameter, 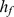 and 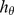 the non-zero auxiliary parameters then the nonlinear differential operators 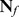 and 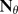 are given by 
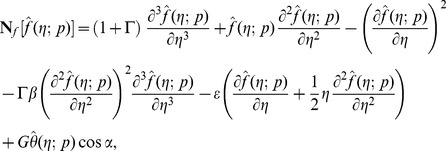
(17)

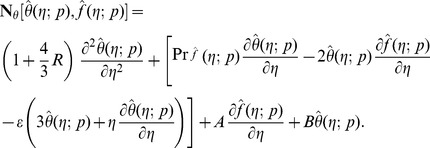
(18)


We have for p = 0 and p = 1 the following equations
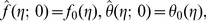
(19)

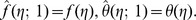



It is noticed that when p varies from 0 to 1 then 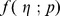 and 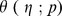 approach from 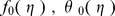 to 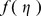 and 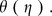 The series of 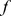 and 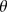 through Taylor's expansion are chosen convergent for p = 1 and thus
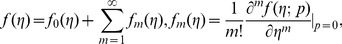
(20)

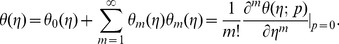
(21)


The resulting problems at 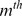 order can be presented in the following forms 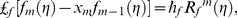
(22)

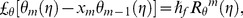
(23)

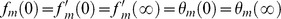


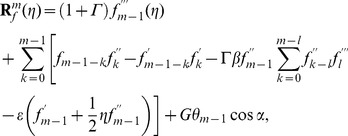
(24)

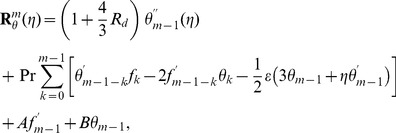
(25)

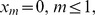


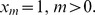



The general solutions 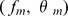 comprising the special solutions 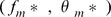 are
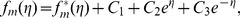
(26)

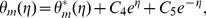
(27)


### Convergence of the homotopy solutions

**Figure 2:**
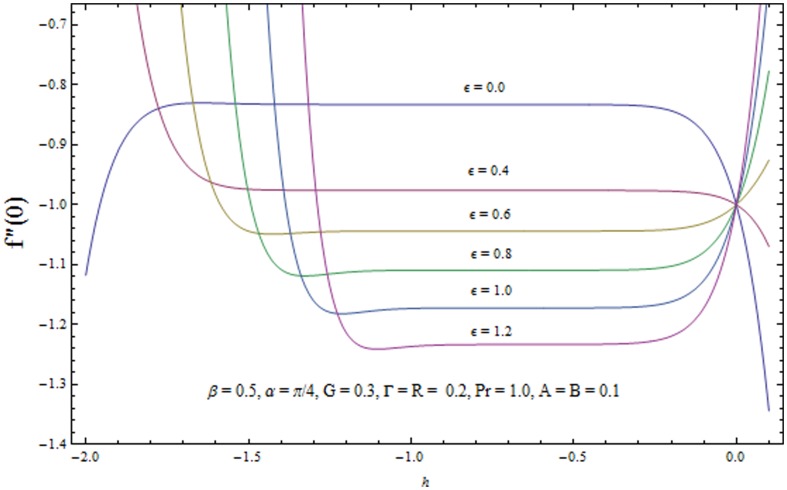
The 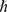-curves for the velocity field.

**Figure 3:**
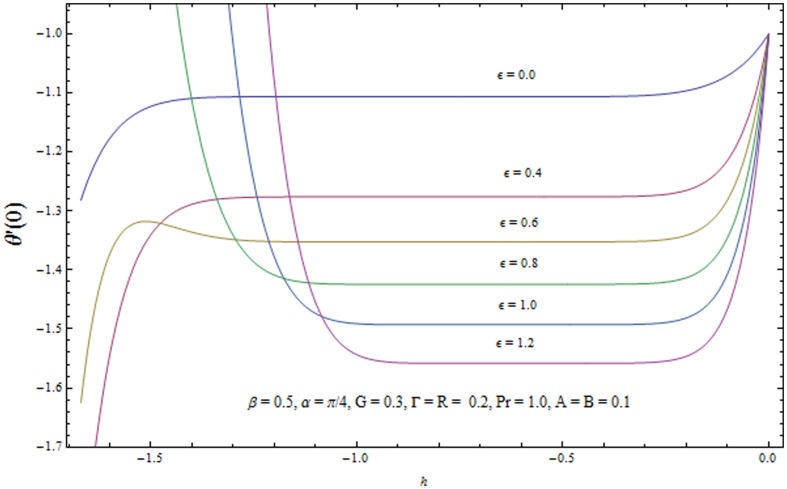
The 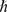-curves for the temperature field.

It is now a well established argument that the convergence of series solutions (22) and (23) depends upon the auxiliary parameters 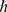. The admissible range of values of 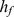 and 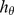 (for some fixed values of parameters) lie along the line segment parallel to 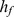 and 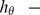 axes. For example in Figs. 2 and 3 the permissible range of values of 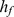 and 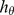 are 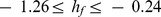 and 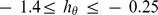 respectively when 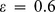. This series solutions converge for the whole region of 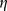 when 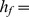
−0.9 and 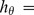
−0.8. Table 1. shows the convergence of HAM solution for different order of approximations. It is clear from this table that 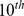 order of approximations are sufficient for convergent solutions up to six decimal place.

**Table 1:** Convergence of series solutions for different order of approximations when α = π/4, β = 0.5, Г = 0.2, R = 0.2, 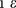 = 0.6, G = 0.3, Pr = 1.0, A = B = 0.1, h–_f_ = −0.8 and h–_e_ = −0.7.

Order of approximation	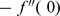	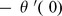
1	1.03515	1.33250
5	1.04402	1.35252
10	1.04401	1.35252
15	1.04401	1.35252
20	1.04401	1.35252
30	1.04401	1.35252

## Results and Discussion

**Figure 4:**
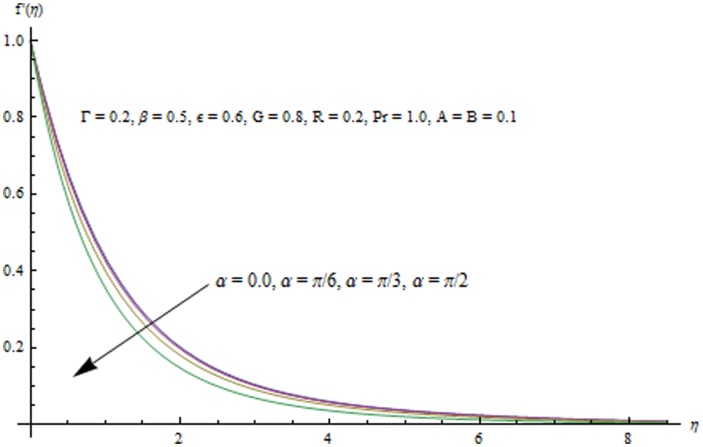
Influence of 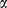 on the velocity field.

**Figure 5:**
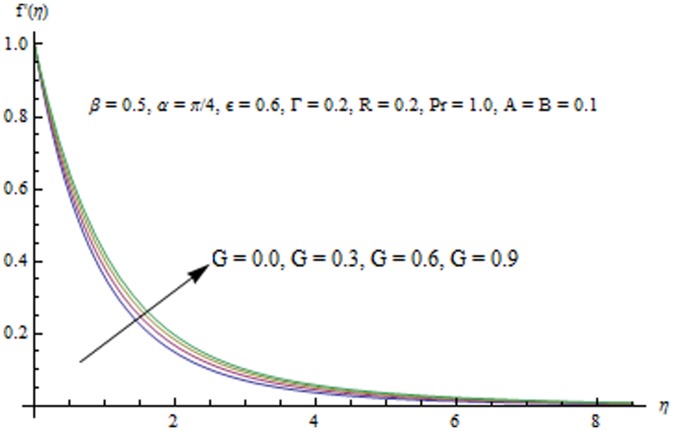
Influence of G on the velocity field.

**Figure 6:**
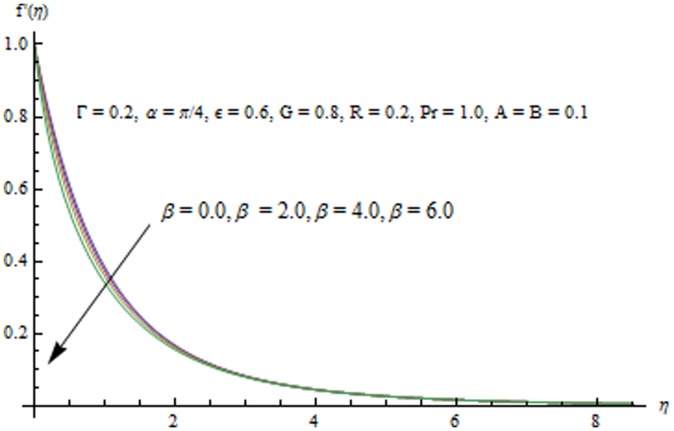
Influence of 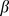 on the velocity field.

**Figure 7:**
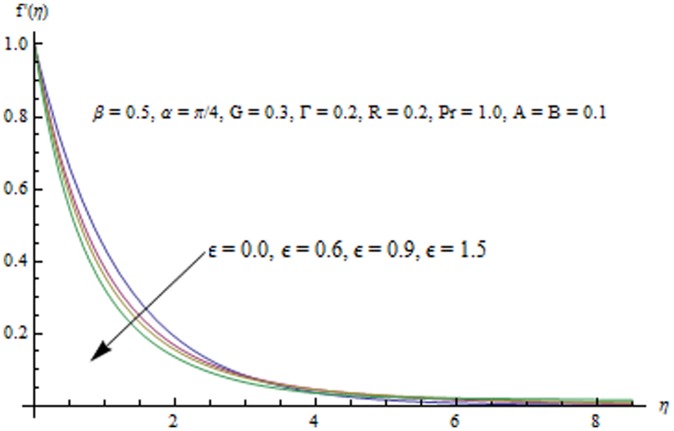
Influence of 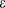 on the velocity field.

**Figure 8:**
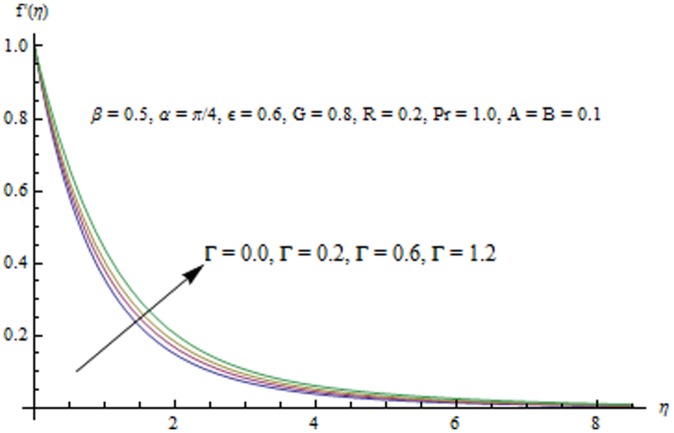
Influence of 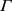 on the velocity field.

**Figure 9:**
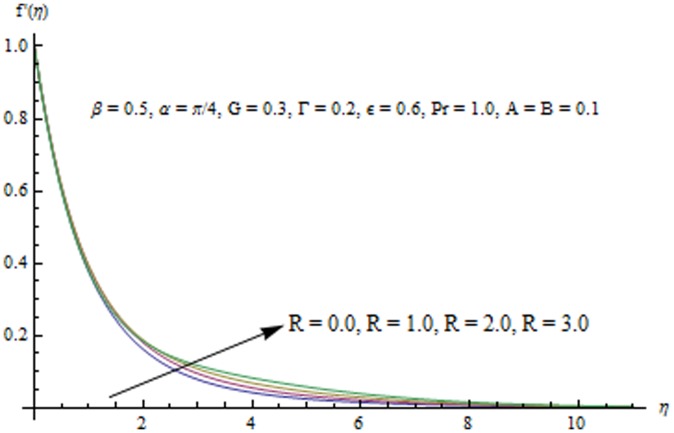
Influence of R on the velocity field.

**Figure 10:**
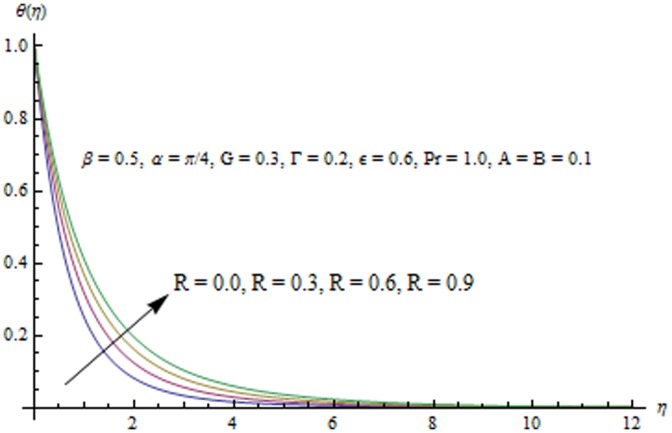
Influence of R on the temperature field.

**Figure 11:**
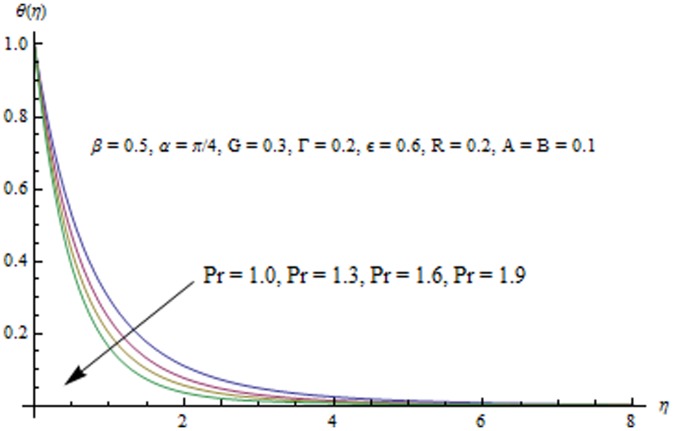
Influence of 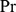 on the temperature field.

**Figure 12:**
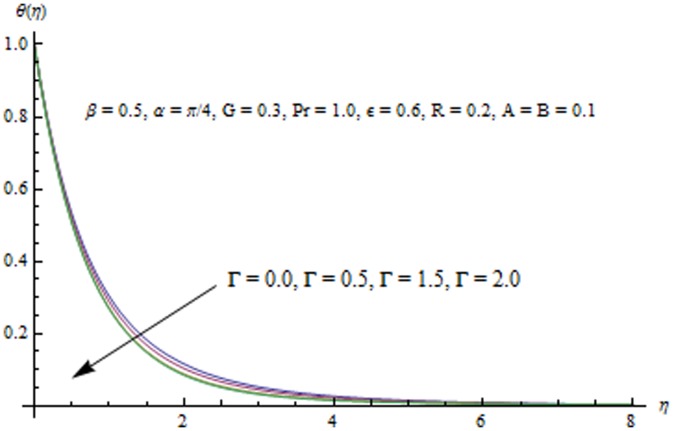
Influence of 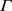 on the temperature field.

**Figure 13:**
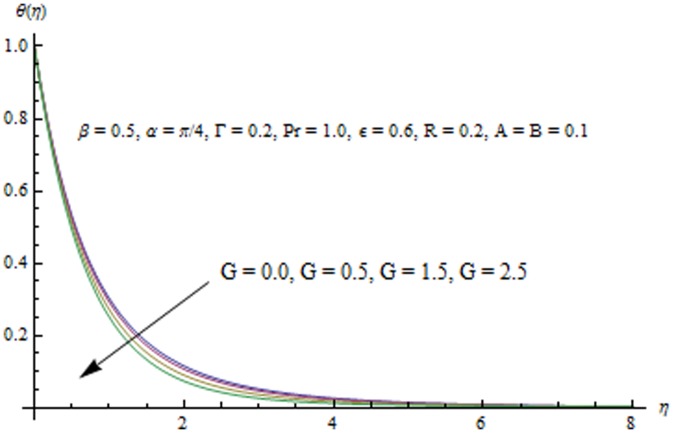
Influence of G on the temperature field.

**Figure 14:**
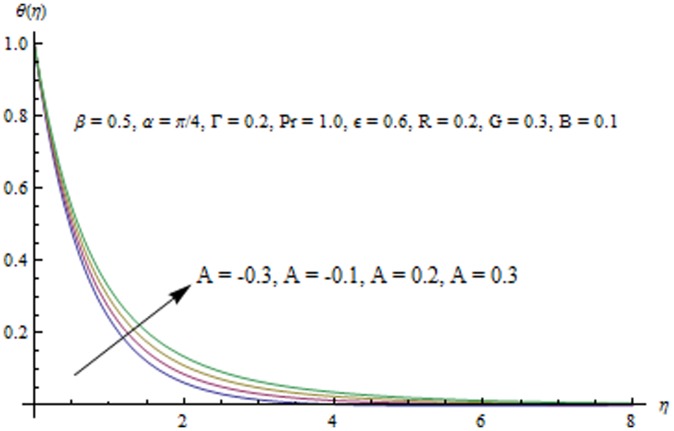
Influence of A on the temperature field.

**Figure 15:**
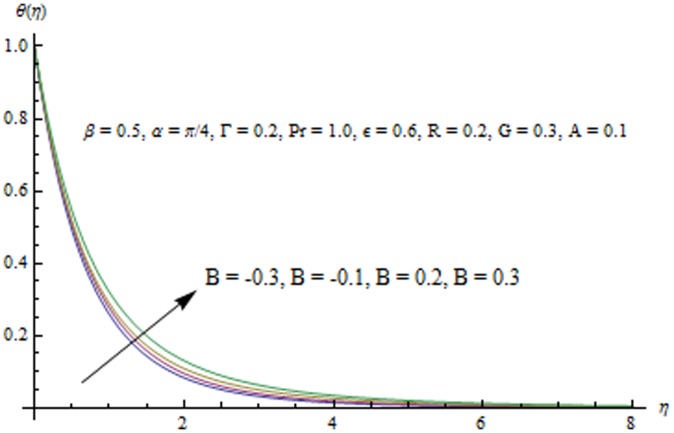
Influence of B on the temperature field.

**Figure 16:**
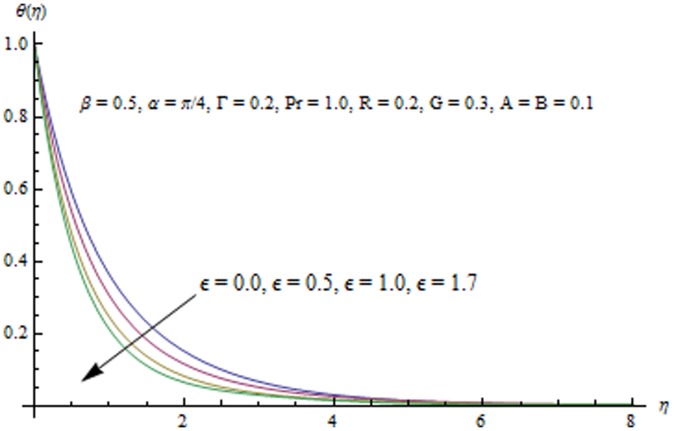
Influence of 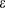 on the temperature field.

This section examines the effects of different physical parameters on the velocity and temperature fields. Hence Figs. (4, 5, 6, 7, 8, 9, 10, 11, 12, 13, 14, 15, 16) are plotted. Fig. 4 elucidates the behavior of inclination angle 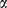 on the velocity and the boundary layer thickness. 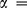
0 shows the corresponding velocity profiles in the case of a vertical sheet for which the fluid experiences the maximum gravitational force. On the other hand when 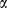 changes from 0 to 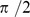 i.e. when the sheet moves from vertical to horizontal direction, the strength of buoyancy force decreases and consequently the velocity and the boundary layer thickness decrease. Fig. 5 indicates that velocity field 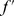 is an increasing function of 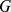. This is because a larger value of 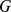 accompanies a stronger buoyancy force which leads to an increase in the 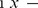 component of velocity. The boundary layer thickness also increases with an increase in 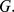 Variation in 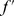 with an increase in 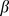 can be seen from Fig. 6. It is noticed that 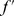 decreases and boundary layer thins when 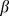 is increased. Influence of unsteady parameter 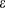 on the velocity field is displayed in Fig. 7. Increasing values of 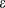 indicates smaller stretching rate in the x - direction which eventually decreases the boundary layer thickness. Interestingly the velocity increases by increasing 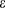 at sufficiently large distance from the sheet. Variation in the x-component of velocity with an increase in the fluid parameter 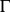 can be described from Fig. 8. In accordance with Mushtaq et al. [25], the velocity field 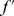 increases with an increase in 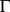.

Radiation effects on the velocity and temperature distributions are perceived from Figs. 9 and 10. An increase in R enhances the heat flux from the sheet which gives rise to the fluid's velocity and temperature. Wall slope of the temperature function therefore increases with an increase in R. Fig. 11 portrays the effect of Prandtl number on the thermal boundary layer. From the definition of 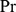 given in Eq. (10), it is obvious that increasing values of 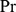 decreases conduction and enhances pure convection or the transfer of heat through unit area. That is why temperature and the thermal boundary layer thickness decrease with an increase in 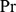. This reduction in the thermal boundary layer accompanies a larger heat transfer rate from the sheet. Temperature profiles for different values of 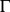 are shown in Fig. 12. It is seen that temperature 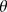 is an increasing function of 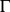. Fig. 13 indicates that an increase in the strength of buoyancy force due to temperature gradient decreases the temperature and the thermal boundary layer thickness. Influence of heat source/sink parameters on the thermal boundary layer are presented in Figs. 14 and 15. As expected the larger heat source (corresponding to A>0 and B>0) rises the fluid's temperature above the sheet. While the non-uniform heat sink corresponding to A<0 and B<0 can contribute in quenching the heat from stretching sheet effectively. Fig. 16 depicts that temperature 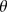 is a decreasing function of the unsteady parameter 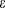.

Table 2 shows comparison of present work with Tsai et al. in a special case. A very good agreement is found between the results of wall temperature gradient. Table 3 shows the effect of embedded parameters on heat transfer characteristics at the wall 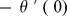. Since in the present case the sheet is hotter than the fluid i.e 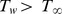 thus heat flows from the sheet to the fluid and hence 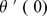 is negative. From this table we observe that with an increase in 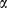, 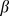 and R the wall heat transfer rate 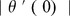 decreases. However it increases when 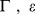
and 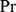 are increased.

**Table 2:** Comparison between numerical solution Tsai et. al. [7] and HAM solution in a special case when 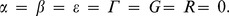

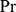	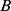	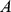	Present study	Tsai et. [7]
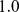	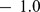	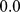	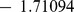	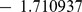
	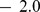	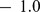	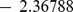	
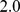	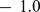	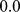	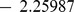	
	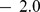	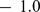	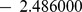	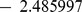

**Table 3:** Values of heat transfer characteristics at wall 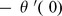 for different emerging parameters when h–_f_ = −0.8 and h–*_θ_*  = −0.7.

α	Г	β	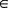	G	R	Pr	A	−(1+ 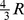 ) 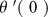
0.0								1.35702
π/6								1.35798
π/3								1.34926
π/4	0.0							1.69881
	0.4							1.72556
	0.7							1.74109
	0.9							1.74984
		0.0						1.71555
		0.5						1.71319
		0.9						1.71114
			0.0					1.10162
			0.4					1.61674
			0.6					1.71319
				0.0				1.69868
				0.5				1.72227
				0.8				1.73515
					0.0			1.54046
					0.3			1.71319
					0.6			2.00303
						1.2		1.91058
						1.5		2017721
						1.9		2049323
							−0.1	1.80783
							0.0	1.76059
							0.1	1.71319

## Conclusions

This article addressed the radiation effects in the unsteady boundary layer flow of Powell-Eyring fluid past an unsteady inclined stretching sheet with non-uniform heat source/sink. Homotopy analysis method (HAM) was used to obtain approximate analytic solutions of the governing nonlinear problem. The important findings are listed below.

1. The strength of gravitational force can be varied by changing the inclination angle 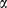 which the sheet makes with the vertical direction. The velocity decreases with an increase in 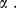

2. Velocity field 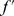 and temperature 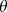 are decreasing function of the unsteady parameter 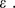

3. Velocity increases and temperature decreases when the fluid parameter 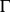 is increased.
4. Increase in the radiation parameter R enhances the heat flux from the plate which increases the fluid's velocity and temperature.
5. The analysis for the case of viscous fluid can be obtained by choosing 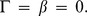 Further the results for horizontal stretching sheet are achieved for 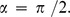
